# Clinical outcomes and risk factors for local failure and visual impairment in patients treated with Ru-106 brachytherapy for uveal melanoma

**DOI:** 10.1016/j.ctro.2025.100939

**Published:** 2025-02-24

**Authors:** L.J. Pors, M. Marinkovic, H.H. Deuzeman, T.H.K. Vu, E.M. Kerkhof, K.M. van Wieringen-Warmenhoven, C.R.N. Rasch, J.C. Bleeker, L.S. Koetsier, J.W.M. Beenakker, G.P.M. Luyten, C.L. Creutzberg, N. Horeweg

**Affiliations:** a Department of Radiation Oncology, Leiden University Medical Center, Leiden, the Netherlands; b Department of Ophthalmology, Leiden University Medical Center, Leiden, the Netherlands; c Department of Radiology, Leiden University Medical Center, Leiden, the Netherlands

**Keywords:** Uveal melanoma, Brachytherapy, Visual acuity, Neoplasm recurrence, Local

## Abstract

**Background and purpose:**

Small uveal melanomas are preferably treated with eye-sparing therapies such as brachytherapy or proton beam therapy. Knowledge of clinical outcomes and risk factors is important for personalized patient counselling.

**Materials and methods:**

Data of all choroidal melanoma patients treated at the Leiden University Medical Center between 2012–2019 with Ruthenium-106 brachytherapy were collected. Time-to-event analyses were performed with the Kaplan-Meier method, risk factors for local failure and visual acuity (VA) decline were identified using Cox proportional hazards models with predefined covariates.

**Results:**

719 patients were included. Median tumour prominence and diameter were 3.8 mm and 11.6 mm, respectively. 52 % of tumours were centrally located, and 19 % were juxtapapillary. Five-year overall survival, eye retention and local failure rates were 83 %, 95 %, and 8 %, respectively. Juxtapapillary location, lower tumour apex doses, and T3-4 tumour stage were significantly associated with local failure. Patients with juxtapapillary tumours had 20 % risk of local failure. Five years posttreatment, 47 % of patients had no functional visual impairment (Snellen VA ≥ 0.5). Lasting functional visual impairment was associated with baseline vision, central tumours, and scleral dose. Patients with central tumours had 75 % risk of mild or worse visual impairment after 5 years.

**Conclusion:**

Ruthenium-106 brachytherapy yields good local control and visual outcomes in patients with choroidal melanoma. Juxtapapillary tumours have a high risk of local failure and alternative therapies should be considered in these patients, if available. Patients with central tumours, and those with decreased pre-treatment vision should be counselled on the risk of visual impairment.

## Highlights

• Local control 5 years after Ru-106 brachytherapy for uveal melanoma was high: 92 %.
• However, local failure is common in juxtapapillary tumours: 20%.
• Lower tumour apex doses, especially < 110 Gy-eq, increase risk of local failure.
• 60% of local failures could be retreated with an eye-sparing therapy.
• Visual impairment risk is 75% in central and 31% in (mid)peripheral tumours.

## Introduction

Uveal melanoma is the most common primary ocular malignancy in adults, and may develop from the choroid (90 %), ciliary body (6 %) or iris (4 %) [1]. These tumours can be treated with enucleation or eye-sparing therapies, such as Ru-106 or I-125 plaque brachytherapy or external beam therapy [1], [2], [3], [4]. Ru-106 has the smallest therapeutic range [5], leading to less toxicity [6], [7].

When counselling patients for Ru-106 brachytherapy, the probabilities of local control, eye preservation, loss of visual acuity (VA) and ocular toxicities are essential. However, discussing these, in addition to treatment procedures, can be overwhelming and challenging due to time restrictions. Knowledge about risk factors for adverse events may help tailor consultations.

Local recurrence probability has been linked to both more and less advanced age [8], [9], [10], male sex [11], lower baseline VA [9], [12], larger tumour size [10], [11], [12], [13], [14], [15], [16], central or juxtapapillary tumour location [8], [12], [16], [17], [18], Bruch’s membrane rupture [14], lower radiation dose [11], [13], [17], and inadequate plaque positioning [19]. As correction for confounding was rarely performed in these studies, it is likely that only a few have relevant clinical relations with local failure. Loss of VA is associated with higher patient age [20], [21], lower baseline VA [10], [19], [20], [22], [23], larger tumour size [10], [19], [20], [21], [22], [23], [24], [25] and tumour location [10], [14], [19], [21], [22], [23], [24], [25]. In many of these studies, correction for confounding was also not performed.

We periodically evaluate outcomes of Ru-106 brachytherapy at our centre [10], [12], [26], [27]. Our latest evaluation (cohort treated in 2004–2011) led to abandoning the use of transpupillary thermotherapy (TTT) in combination with Ru-106, which yielded similar efficacy and improved visual outcomes [10]. Here, we evaluate oncological and ocular outcomes of patients treated in 2012–2019 to refine indication criteria for Ru-106 and enable personalized counselling through identification of risk factors for local recurrence and VA loss.

## Materials and methods

### Patients and data collection

Patients treated in 2012–2019 with Ruthenium-106 brachytherapy at Leiden University Medical Center (LUMC) for a choroid or ciliary body melanoma were included. Those treated with local primary therapies other than Ru-106 alone, and those metastasized at diagnosis were excluded.

Data was retrospectively collected from electronic patient files after ethical approval by our local IRB board (registration number nWMO-D4-2022-018). Tumour location, proximity to the optic disc and tumour response to treatment (based on size and fluorescence angiography (FAG) activity) were reviewed and scored as previously described [26], [28]. Local failure was defined as tumour growth of > 0.6 mm in prominence [29], and/or clinically significant growth at the lateral tumour border, and/or remaining active tumour tissue requiring therapeutic intervention. Overall survival data was collected from the Dutch Personal Records Database. Toxicity was scored using the Common Terminology Criteria for Adverse Events (CTCAE V5.0) [30].

### Treatment protocol

The diagnostic work-up of uveal melanoma consisted of assessment of best-corrected VA (Snellen), slit-lamp examination, fundoscopy, ocular ultrasound, FAG, gonioscopy or ocular MRI[31] (when indicated), and distant metastasis screening using abdominal ultrasound and chest X-ray. Tumours ≤ 7 mm in prominence (including the sclera) and ≤ 16 mm in diameter were considered eligible for Ru-106. As proton beam therapy was not available in the Netherlands until 2020 [28], larger tumours were sometimes treated with Ru-106 as well, after shared decision-making.

Planned tumour apex dose was 130 Gy-equivalent. Dose rate was standardized to 100 Gy/24 h as previously described [26], [32]. A minimal scleral dose of 300 Gy-equivalent was applied until 2014. The maximum acceptable deviation from the calculated application time was 5 %.

Under general anaesthesia, optimal plaque location was determined using transillumination and/or indentation and fundoscopy. A dummy plaque was positioned and checked, whereafter the treatment plaque was placed with ≥ 1 mm margin. Eye muscles were temporarily disinserted if needed. Plaque removal was performed under local anaesthesia.

Follow-up was performed telephonically two weeks after plaque removal and after 3 and 7 months in our outpatient clinic, then biannually until 3 years after therapy, and yearly thereafter. Follow-up visits consisted of VA assessment (Snellen), intra-ocular pressure measurement, slit lamp examination, fundoscopy, ocular ultrasound, optical coherence tomography and FAG, as indicated. Distant metastasis screening was performed biannually using abdominal ultrasound.

### Stateistics

Length of follow-up was calculated using the reverse Kaplan-Meier method [33]. Time to event data were truncated at five years to account for the cohorts follow-up time difference and analysed using the Kaplan-Meier methodology; differences between groups were assessed using the log-rank test [34]. Patients were censored at last moment known to be alive, last visit to any doctor and last visit to the LUMC outpatient clinic for overall survival, distant metastases, and ocular outcome analyses, respectively. Risk factors for local recurrence and persistent loss of VA below 0.1 and below 0.5 (Snellen) were identified using a Cox proportional hazards model with predefined covariates based on the literature and expertise from our ocular oncologists. Missing VA data, binned per year, were imputed within cases using linear interpolation between available measurements. Hierarchical clustering with Euclidean distances and Ward.D2 linkage using the R (version 4.4.1) ComplexHeatMap package (https://www.r-project.org/;) [35], [36] was performed to visualise the course of VA. Crude rates of the highest toxicity grades during the entire follow-up time were reported with censoring at re-irradiation or enucleation.

## Results

### Cohort

Characteristics of the total cohort of 719 patients are summarized in Table 1. Median tumour prominence and diameter were 3.8 and 11.6 mm, respectively. 43 (6 %) and 27 (4 %) tumours were > 7 mm in prominence and > 16 mm in diameter, respectively. Most tumours were primarily choroidal (676, 94 %). 377 tumours (52 %) were centrally located, while 136 (19 %) were juxtapapillary. Median prominences and diameters of juxtapapillary tumours were 3.5 mm (1.8–7.3) and 10.5 mm (6.5–16.3), respectively, and these were treated with the notched COB plaque (n = 96, 71 %), or with regular CCD (n = 12), CCB (n = 13) or CCA plaques (n = 15).

**Table 1:** Baseline characteristics.

**Included patients**	719
**Age (years)**	66 (20–93)
**Sex (female)**	382 (53 %)
**Affected eye (right)**	374 (52 %)
**Diabetes**	107 (15 %)
**Hypertension**	297 (41 %)
**Tumour prominence (mm)**	3.8 (1.8–8.9)
**Tumour diameter (mm)**	11.6 (3.6–19.0)
**T-stage (AJCC 8^th^ed.)** T1 T2 T3 T4*	200 (28 %) 398 (55 %) 120 (17 %) 1 (0.1 %)
**Tumour location** Central Midperipheral Peripheral	377 (52 %) 255 (36 %) 87 (12 %)
**Eye structures involved by tumour∼** Choroid Ciliary body Iris Anterior chamber	695 (97 %) 55 (8 %) 23 (3 %) 33 (5 %)
**Extrascleral tumour extension**	9 (1 %)
**Juxtapapillary location** (<1 disc diameter from ocular nerve)	135 (19 %)
**Tumour apex dose (equivalent, Gy)**	129 (81–209)
**Scleral dose (equivalent, Gy)**	370 (182–1701)
**Plaque type^+^** Notched Regular	99 (14 %) 620 (86 %)

Continuous variables: median (range).

*In one lesion, the basal diameter of a flat tumour extension was difficult to measure and estimated at 18 mm, classifying it as a T4 tumour, but was treatable with Ru-106.

∼Tumours could involve more than one eye structure per patient.

+ CCB(active diameter 18.2 mm): 239 (33 %), CCD (15.9 mm): 227 (32 %), CCA (13.3 mm): 148 (21 %), COB (notched, 17.8 mm): 96 (13 %), CCX (9.6 mm): 6 (1 %), CIA (notched, 13.3 mm): 3 (0.4 %), Eckert & Ziegler, BEBIG, Berlin, Germany). The notched CIA plaques were used for three anterior tumours.

In the complete cohort, median tumour apex and scleral doses were 129 Gy-equivalent (81–209) and 370 (182–701) Gy-equivalent, respectively. In 3 patients, plaque position was changed during treatment, extending the target area to include a flat tumour extension. In 14 (2 %) and 29 (4 %) patients, the applicator was removed below 95 % or above 105 % of the calculated application time, respectively. 14 (2 %) and 169 (24 %) patients received < 110 Gy-equivalent to the tumour apex and < 300 Gy-equivalent to the sclera, respectively.

### Oncological outcomes

Median follow-up times for overall survival (through national registry), distant metastasis (follow up by any doctor) and ocular outcomes (follow up at LUMC) were 7.7 years, 5.2 years and 4.7 years, respectively. 5-year overall survival and probability of metastasis were 83 % (95 % C.I. 80 %–85 %) and 12 % (95 % C.I. 9 %–14 %), respectively. 92 (13 %) patients developed metastases, located only intrahepatic in 71 (77 %), intra- and extrahepatic in 17 (18 %), and only extrahepatic in 4 (4 %) patients. Extrahepatic lesions are detailed in Supplementary Table 1.

### Tumour response

**Fig. 1:**
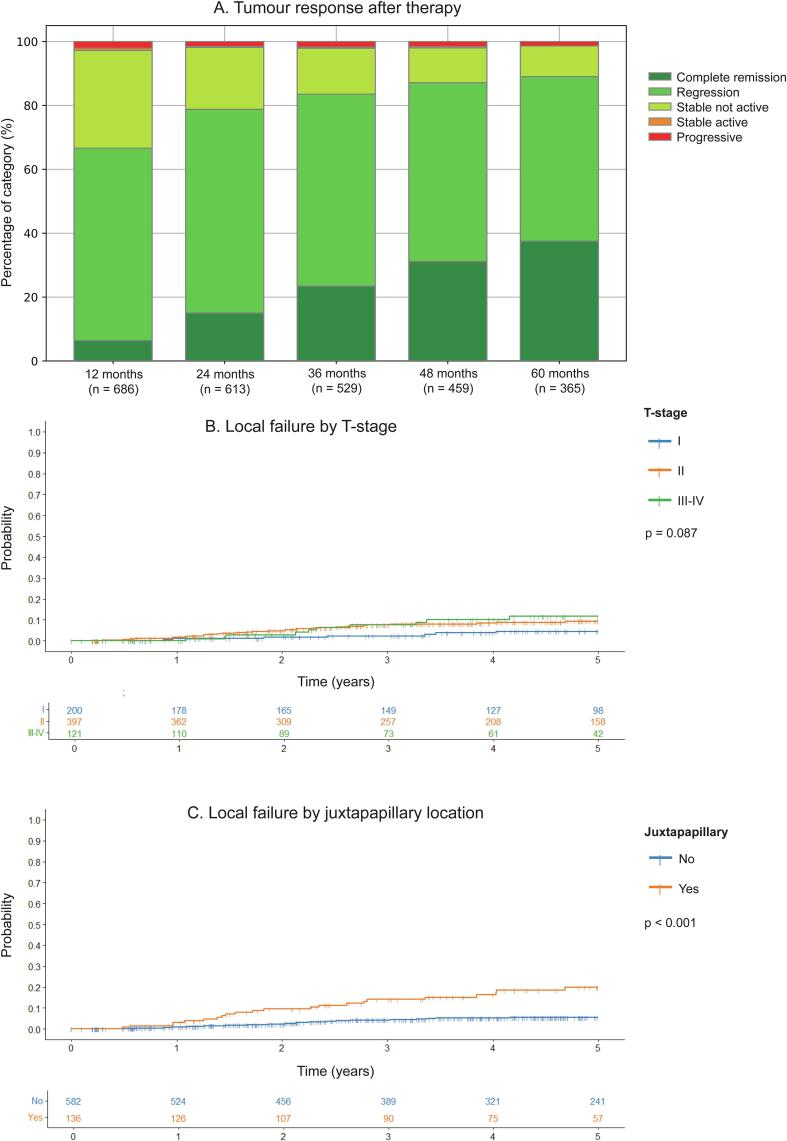
Title: Tumour response status after Ruthenium-106 brachytherapy. Caption: A) Evolution of tumour response during follow-up. The bars at each time point represent the distribution of the patients still in follow-up at that time point across the 5 different categories of response. Patients with local failures (having stable active or progressive tumours) were retreated. The tumour response to salvage treatment is used for the subsequent time points. Patients who underwent enucleation were usually referred back to their local ophthalmologist and were consequently lost to follow-up shortly thereafter. In 15 cases, apparent tumour growth was, in hindsight, not true progression but attributable to tumour growth in the waiting time before the start of treatment. These were not retreated and were not viewed as local failures in the analyses. B) Impact of tumour stage on local failure, log rank test C) Impact of juxtapapillary tumour location on local failure, log rank test.

Response to therapy is shown in Fig. 1A. One year after therapy, 6 % of tumours were in complete remission, 60 % in partial remission, 31 % stable and inactive, 1 % stable but active and 2 % progressive.

84 secondary interventions were performed after Ruthenium-106 brachytherapy in 78 patients either for local failure (n = 52 of 719, 7.2 %) or tumour toxicity (n = 26 of 719, 3.6 %). 34 (44 %), 31 (40 %), 14 (18 %), 3 (4 %) and 1 (1 %) patients were treated with TTT, enucleation, repeat Ruthenium-106 brachytherapy, endoresections and proton beam therapy, respectively. The 5-year enucleation probability for any reason was 5 % (95 % C.I. 3 %–7 %). Reasons for enucleation are detailed in Supplementary Table 2. In seven patients, the ocular oncologist advised a secondary treatment for toxicity (n = 4) or local failure (n = 3), but this was not performed after shared decision-making. At five years, including any salvage treatments, 38 % of all tumours were in complete remission, 51 % in partial regression, 10 % stable and inactive, none were stable but active, and 1 % was progressive.

**Supplementary Figure 1:**
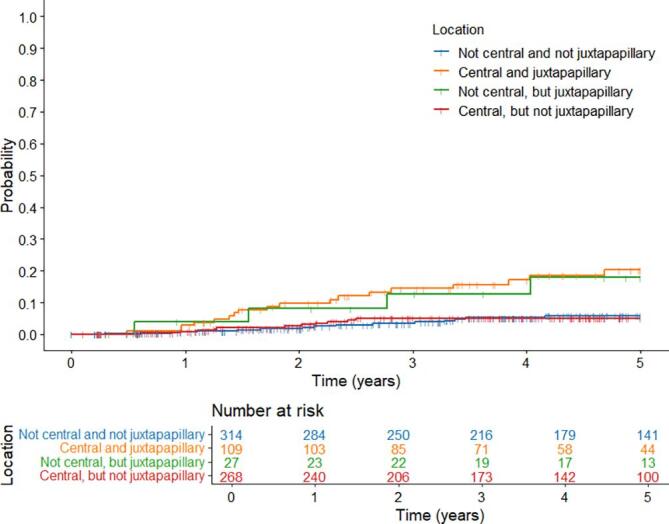
The impact of central and justapapillary location on the risk of local failure in the first five years after therapy.

In the complete cohort, the 5-year local failure rate was 8 % (95 % C.I. 6 %–11 %). The difference in local failure probability did not reach statistical significance between T-stages (Fig. 1A), but did for juxtapapillary localisation (20 %, 95 % C.I. 12 %–27 % versus 6 %, 95 % C.I. 3 %–8 %, p < 0.001, Fig. 1B). Juxtapapillary tumours treated with regular plaques had a numerically, but statistically not significant, lower 5-year local failure probability (9 %, 95 % C.I. 0 %–19 %) than those treated with notched plaques (24 %, 95 % C.I. 15 %–34 %, p = 0.090). Juxtapapillary tumours that were respectively centrally and (mid)peripherally located had five-year local failure risks of 20 %(95 % C.I. 11 %–18 %) and 18 %(95 % C.I. 0 %–33 %), while these risks were 5 % (95 % C.I. 2 %–8 %) and 6 % (95 % C.I. 3 %–9 %) in centrally and (mid)peripherally located non-juxtapapillary tumours, respectively (Supplementary Fig. 1).

Of 52 local failures, 30 (58 %) were margin and 22 (42 %) in-field failures. Patients with tumour apex doses < 110 Gy-equivalent had large prominence tumours (median 7.6 mm), and a significantly higher 5-year local failure rate (32 %, 95 % C.I. 1 %–63 % vs. 8 %, 95 % C.I. 6 %–10 %, p = 0.014) than those treated with ≥ 110 Gy-equivalent. Scleral doses < 300 Gy-equivalent did not affect 5-year local failure rates (6 %, 95 % C.I. 3 %–9 % vs. 10 %, 95 % C.I. 7 %–13 %, p = 0.19).

After correction for confounding, juxtapapillary location was strongly associated with local failure (HR 4.906, 95 % C.I. 2.666–9.028, p < 0.001, Supplementary Table 3), as were a lower equivalent tumour apex dose (HR 0.971, 95 % C.I. 0.945–0.997, p = 0.032) and T3-4 tumour stage (compared to T1, HR 4.541, 95 % C.I. 1.097–9.106, p = 0.033).

### Visual acuity

**Fig. 2:**
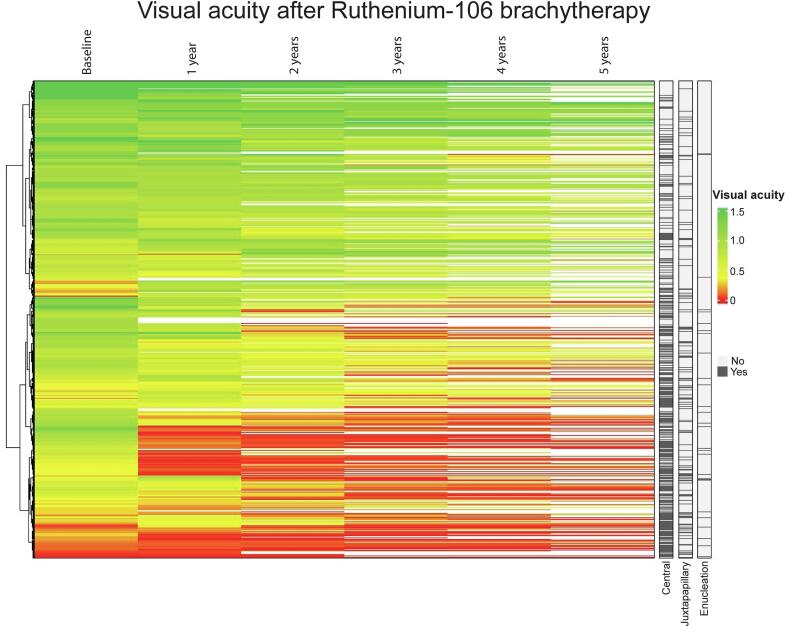
Title: Visual acuity after Ruthenium-106 brachytherapy. Caption: Course of visual acuity after Ruthenium-106 brachytherapy.

At baseline, 78 %, 12 %, 7 % and 3 % of patients had no (ICD-11: Snellen VA ≥ 0.5) [37], mild (VA 0.3–0.5), moderate (VA 0.1–0.3) or severe (VA < 0.1) functional visual impairment, respectively (Fig. 2). After brachytherapy, visual impairment gradually progressed to respectively 47 %, 9 %, 13 % and 31 % at five years. 80 % of retreated patients had any visual impairment (VA < 0.5) after five years. VA decline was significantly larger for the affected than the unaffected eye (median change −0.3 vs. 0, Mann Whitney U p < 0.001). VA improvement after treatment was observed in 186 (27 %) and 52 (16 %) patients at 1 and 5 years posttreatment, respectively.

**Fig. 3:**
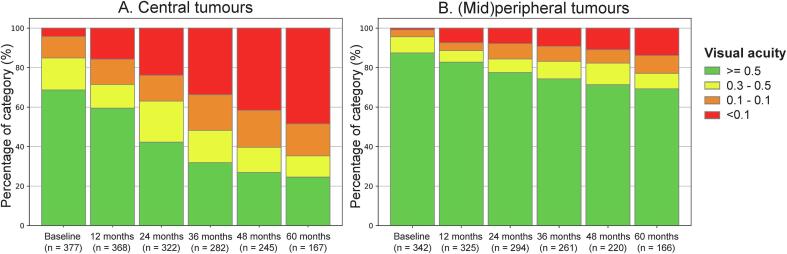
Title: Impact of tumour location on visual acuity. Caption: VA after Ruthenium-106 brachytherapy in A) central tumours and B) (mid)peripheral tumours.

The 5-year actuarial estimates of lasting VA below 0.1 and 0.5 were 32 % (95 % C.I. 28 % – 36 %) and 54 % (95 % C.I. 50 % – 58 %), respectively. In multivariable analyses, pretreatment VA, central tumour localisation and scleral dose had a significant impact on the risk of a lasting posttreatment VA below 0.1 and below 0.5, while age at diagnosis and T-stage were not significantly associated (Table 2). Fig. 3 illustrates this effect of central tumour localization on VA. After five years, 75 % of patients with central tumours had a VA < 0.5, while this occurred in 31 % of patients with (mid)peripheral tumours.

**Table 2:** Risk factors for a lasting loss of VA in the first five years after Ruthenium-106 brachytherapy.

	**Covariate**	**Hazard ratio**	**Confidence interval**	**p-value**
**Visual acuity below 0.1**	Visual acuity before treatment (Snellen)	0.280	0.188–0.418	<0.001
	Central tumour location	3.476	2.418–4.998	<0.001
	Scleral dose (Gy equivalent)	1.002	1.001–1.002	<0.001
	Age at diagnosis	1.011	0.999–1.022	0.073
	T stage III or IV	0.972	0.637–1.484	0.896
				
**Visual acuity below 0.5**	Visual acuity before treatment (Snellen)	0.232	0.172–0.314	<0.001
	Central tumour location	2.926	2.271–3.769	<0.001
	Scleral dose (Gy equivalent)	1.001	1.001–1.002	<0.001
	Age at diagnosis	1.004	0.996–1.012	0.374
	T stage III or IV	0.972	0.706–1.338	0.862

Results from multivariable analyses with correction for treatment year. These covariates were chosen based on the shown correlation of pretreatment visual acuity [10], [19], [20], [22], [23], central tumour location [10], [14], [19], [20], [21], [22], [23], [25], tumour height [10], [19], [20], [22], [23], [24] (which is, as brachytherapy treatment is dosed to the tumour apex, strongly correlated with scleral dose), patient age [20], [21], and tumour height [10], [19], [20], [22], [23], [24] and diameter [20], [21], [24] (which are both represented in the T-stage), combined with their biologically plausible connection with risk of visual acuity loss.

### Toxicity

Posttherapeutic toxicities are summarized in Table 3. In the first three months after therapy, 265 (37 %) patients experienced pain. Of these, 13 had developed scleritis. Scleral melting was found in six patients, two of which grade 4, leading to an enucleation. 15 % of patients reported, usually low-grade, late pain. Of 606 patients who had not undergone cataract surgery, 300 (50 %) had cataract before treatment, which increased to 417 (58 %) patients in follow-up, leading to cataract surgery in 122 (20 %) patients. Corrected for the presence of glaucoma at baseline, ciliary body tumours had a higher glaucoma risk (HR 4.562, 95 % C.I. 2.354–8.843, p < 0.001). Optic nerve disorder was reported in 80 (11 %) patients and was associated with juxtapapillary tumour location after correction for baseline disease (HR 5.108, 95 % C.I. 3.124–8.351, p < 0.001). Maculopathy occurred in 46 patients (6 %) at baseline, and in 254 (35 %) patients during follow-up, and was treated with intravitreal anti-VEGF or corticosteroid injections, or retinal laser coagulation, upon indication. After correction for baseline maculopathy, tumour location and size had no statistically significant effect on maculopathy risk. Overall, 181 (25 %) patients were treated with intravitreal anti-VEGF injections.

**Table 3:** Treatment-related toxicities after Ruthenium-106 brachytherapy (n = 719).

Toxicity	Baseline	Follow-up				
	Any grade (n (%))	Any grade (n (%))	Grade 1	Grade 2	Grade 3	Grade 4
**Acute toxicity (< 3 months after therapy)**						
Acute pain		265 (36.9 %)	177 (24.6 %)	86 (12.0 %)	2 (0.3 %)	0 (0 %)
Scleritis		13 (1.8 %)	3 (0.4 %)	8 (1.1 %)	1 (0.1 %)	1 (0.1 %)
**Late toxicity (≥3 months after therapy)**						
Late pain		106 (14.7 %)	76 (10.6 %)	27 (3.8 %)	3 (0.4 %)	0 (0 %)
Scleral melting∼		6 (0.8 %)	4 (0.6 %)	0 (0 %)	0 (0 %)	2 (0.3 %)
Cataract (n = 606)*	300 (49.5 %)	417 (68.8 %)	231 (38.1 %)	50 (8.3 %)	7 (1.2 %)	129 (21.3 %)
Glaucoma	45 (6.3 %)	109 (15.2 %)	32 (4.5 %)	69 (9.6 %)	1 (0.1 %)	7 (1.0 %)
Optic nerve disorder	3 (0.4 %)	80 (11.1 %)	20 (2.8 %)	14 (2.0 %)	14 (2.0 %)	32 (4.5 %)
Maculopathy	46 (6.4 %)	254 (35.3 %)	33 (4.6 %)	75 (10.4 %)	85 (1.8 %)	61 (8.5 %)

* Patients with pseudophakic eyes at baseline were excluded from the cataract analyses, leading to n = 606.

∼ Scleral doses in patients with scleral melting ranged between 672–1190 Gy-equivalent.

## Discussion

In this cohort study, we reported clinical outcomes after Ru-106 brachytherapy for uveal melanoma. Local control (92 %) and eye preservation rates (95 %) were high, and most patients retained functional vision. We identified subgroups at substantial risk of local failure: those with juxtapapillary or T3-4 tumours, and those treated with low apex doses, and of poor visual outcomes: those with poor baseline VA or central tumours, and those treated with a high scleral dose.

### Local control

At five years, the local failure probability was 8 %, compared to 1–17 % in the literature [8], [10], [17], [19], [38], [39], [40], [41], [42] and 5 % in our previous evaluation of a cohort with less juxtapapillary and T3-4 tumours [10]. Local recurrence risk has been associated with patient characteristics [8], [9], [38], tumour size and progression [10], [12], [13], [14], [15], [16], [38]), tumour location [8], [12], [16], [17], [18], tumour-related toxicity [9], [12], and treatment characteristics [13], [17], [19], [38]. Patient and treatment characteristics and tumour size and location were confirmed as independent factors in five studies that corrected for confounding [8], [10], [14], [17], [38]. We identified a more advanced tumour stage, lower tumour apex dose and juxtapapillary tumour location as significant independent risk factors for local failure. Tumour stage incorporates tumour prominence, diameter and local extension, all established risk factors for recurrence [10], [13], [14], [15], [16], [38]. Lower tumour [13], [17], [38] and scleral [13] dose have been associated with recurrence risk before. As the therapy is dosed to the tumour apex, associations between scleral dose and recurrence are confounded by tumour height. Accordingly, we observed that tumours treated with an apex dose < 110 Gy-equivalent did, but those treated with scleral doses < 300 Gy-equivalent did not have a higher risk of local failure.

The association between juxtapapillary location and local failure has been described before [12], [17] and is attributable to plaque edge placement difficulties and risk of plaque tilt. In our study, patients with juxtapapillary tumours had a 20 % 5-year local failure rate, compared to 7.3 % in a cohort study of 865 parapapillary tumours treated with proton beam therapy (PBT) [43], advocating this alternative treatment for juxtapapillary tumours. Interestingly, local failure risk was numerically (though not significantly) lower in juxtapapillary tumours treated with regular plaques (which are used in patients wherein a smaller portion of the optic disk circumference is affected). Juxtapapillary location, both in central and midperipheral tumours, correlated with a higher risk of local failure, while both central and midperipheral non-juxtapapillary tumours had low local failure risks. This is consistent with the findings of Grajewski et al. of worse local control in central juxtapapillary tumours (68 %) than in central, non-juxtapapillary tumours (86 %) [44].

Salvage treatment after local failure consisted of eye-sparing therapies in 60 % of patients and of enucleation in 40 %. The eye retention probability after Ruthenium-106 brachytherapy at five years was 95 %, compared to 96 %–99.4 % in the literature [10], [17], [19], [42]. As PBT is available in the Netherlands since 2020, we currently treat less high-risk tumours with Ru-106 brachytherapy and thus expect a higher eye retention rate.

### Visual acuity

Five years posttreatment, 47 % of patients had no functional visual impairment, compared to 39–67 % [20], [21], [22], [25] in the literature. The five-year rate of severe functional impairment in our cohort was 31 %, and was 18 %–37 % in the literature [20], [21], [22], [25].

Factors reported to be associated with VA loss are patient characteristics [20], [21], tumour size [10], [19], [20], [21], [22], [23], [24], tumour location [10], [14], [19], [20], [21], [22], [23], [24], [25], tumour-related toxicity [10], [19], [20], [22], [23] and treatment characteristics [25]. These were corroborated after confounding correction in eight studies [10], [12], [19], [20], [21], [22], [23], [25], but many articles excluded enucleated eyes from VA analyses. We identified central tumour location, lower pre-treatment VA, and higher scleral dose as significant independent risk factors for lasting severe (VA < 0.1) and mild (VA < 0.5) visual impairment. Tumour location was of particular relevance, as 75 % of the patients with central and 31 % of those with (mid)peripheral tumours had a VA below 0.5 after five years. This effect is attributable to unavoidable high macular doses. The higher risk for patients receiving a higher scleral dose, independent from tumour location, has not been described before. However, a larger tumour height (which, as brachytherapy is dosed to the tumour apex, leads to a higher scleral dose) has been attributed to VA loss [10], [19], [20], [22], [23], [24]. As scleral dose had no impact on local tumour control but did impact visual impairment risk, a minimum dose was rightfully abandoned in our centre in 2014. Patients with these risk factors should be counselled on their risk of posttreatment visual impairment.

### Toxicity

2 % of patients experienced scleritis, compared to 2.4 % in a LUMC cohort of iridociliary tumours [26]. Scleral necrosis was found in 1 % of patients, compared to 2 % in the literature [23]. Cataract rates in the literature ranged between 4–50 %, compared to 69 % including and 31 % excluding subclinical cataracts in our cohort. Glaucoma rates were comparable to the literature (15 % vs 0–19 %) and glaucoma incidence was correlated with tumour ciliary body involvement, possibly through higher risks of rubeosis, pigment dispersion, and a narrower iridocorneal angle. The incidence of optic nerve disease in the literature is 2 % – 33 % [9], [10], [11], [14], [23], [40], [41], [45], compared with 11 % in our cohort. Maculopathy definitions and incidences in the literature vary, leading to reported incidences ranging from 13 %–55 % [9], [11], [14], [23], [40], [41], [45]. We defined maculopathy as macular retinopathy or oedema [46], resulting in an incidence of 35 %, which was not associated with a central tumour location.

### Clinical implications

The findings from this study can be used for personalized patient counselling and to refine indications for Ruthenium-106 brachytherapy. Ru-106 brachytherapy without TTT is safe and effective in small to intermediate-sized tumours, but should be avoided in patients with juxtapapillary tumours if another treatment modality like proton therapy is available. Additionally, a sufficient apex dose of at least 110 Gy-equivalent seems important for tumour control, especially in high tumour stages. Patients with (mid)peripheral tumours can be reassured of their high chance of retaining functional vision after Ru-106 brachytherapy. In contrast, patients with central tumours should be counselled on the high probability of mild or worse posttreatment visual impairment.

### Strengths and limitations

Important strengths of this study are its cohort size, uniformity of treatment and long follow-up. Additionally, patients who develop uveal melanoma metastases after the standard follow-up of five years are often referred to the medical oncology department of LUMC, leading to a robust follow-up for metastasis. Fundus and ultrasound imaging were reviewed for tumour location and treatment toxicity assessment, when relevant. Lastly, we corrected for confounding in the regression analyses.

Inevitably, this paper has its limitations. The prospective study database was supplemented retrospectively, leading to registration bias. The treatment protocol was changed in 2014 to abandon the minimum scleral dose of 300 Gy, possibly leading to temporal bias, for which we corrected in the multivariate analyses. As we used data from the Dutch Personal Records Database, we were able to achieve a long follow-up time for overall survival but had no access to causes of death. Lastly, treatment centres differ, among others, in eligibility criteria (which are dependent on availability of other treatment options), prescription dose, the use of dose rate correction (performed in two institutes to our knowledge), and treatment margins [47]. This should be taken into account when extrapolating our results.

## Conclusion

This study shows that Ruthenium-106 brachytherapy treatment for uveal melanoma resulted in high local control and eye retention rates and a favourable toxicity profile and visual outcomes. However, juxtapapillary tumours should preferably be treated with an alternative treatment method due to the high local failure rates found for these tumours. While visual impairment is relatively rare in patients with (mid)peripheral tumours, this affects the majority of those with central tumours. These outcomes can be used to refine indications for Ruthenium-106 brachytherapy and personalise patient counselling.

**Funding information**

This research was funded in part by a grant from the Dutch Cancer Society.

**Commercial relationships disclosures**

Our institute receives research support from RaySearch Laboratories and 10.13039/100004320Philips Healthcare. CRA, 10.13039/100020894NAH and JWB receive grant support from 10.13039/100007210Varian Medical Systems.

## CRediT authorship contribution statement

**L.J. Pors:** Conceptualization, Methodology, Validation, Formal analysis, Investigation, Data curation, Writing – original draft, Visualization, Project administration. **M. Marinkovic:** Conceptualization, Resources, Data curation, Writing – review & editing. **H.H. Deuzeman:** Data curation, Writing – review & editing. **T.H.K. Vu:** Resources, Writing – review & editing. **E.M. Kerkhof:** Resources, Writing – review & editing. **K.M. van Wieringen-Warmenhoven:** Conceptualization, Methodology, Resources, Writing – review & editing, Supervision, Funding acquisition. **C.R.N. Rasch:** Conceptualization, Methodology, Resources, Writing – review & editing, Supervision, Funding acquisition. **J.C. Bleeker:** Resources, Writing – review & editing. **L. Koetsier:** Resources, Writing – review & editing. **J.W.M. Beenakker:** Conceptualization, Methodology, Writing – review & editing. **G.P.M. Luyten:** Resources, Writing – review & editing. **C.L. Creutzberg:** Conceptualization, Methodology, Resources, Writing – review & editing. **N. Horeweg:** Conceptualization, Methodology, Formal analysis, Data curation, Writing – review & editing, Visualization, Supervision, Project administration.
