# Supplementary material for: Clinical outcomes and risk factors for local failure and visual impairment in patients treated with Ru-106 brachytherapy for uveal melanoma
Source: Clin Transl Radiat Oncol. 2025 Feb 24;52:100939. doi: 10.1016/j.ctro.2025.100939 (PMC11919603; doi:10.1016/j.ctro.2025.100939)
Supplement: Supplementary Data 2 [file mmc2.docx]

*Supplementary Table 1: Extrahepatic metastases*

| Lungs | 8 (38%) |
| --- | --- |
| Lymph nodes | 7 (33%) |
| Bone | 7 (33%) |
| Skin | 4 (19%) |
| Spleen | 2 (10%) |
| Stomach | 2 (10%) |
| Adrenal gland | 2 (10%) |
| Peritoneum | 2 (10%) |
| Brain | 1 (5%) |
| Axilla | 1 (5%) |
| Bladder | 1 (5%) |
| Omentum | 1 (5%) |
| Mesentery | 1 (5%) |
| Muscles | 1 (5%) |

*Locations of lesions in patients (n=21) with extrahepatic metastases at the time of diagnosis of metastasized disease. In patients with only extrahepatic lesions, these were in the lungs (n=3), lymph nodes (n=2), skin (n=1), stomach (n=1), peritoneum (n=1) and adrenal gland (n=1).*

*Supplementary Table 2: Reasons for enucleation*

| Local failure | 21 (66%) |
| --- | --- |
| Pain/ discomfort | 5 (16%) |
| Secondary glaucoma | 5 (16%) |
| Retinal detachment | 2 (6%) |
| Rubeosis | 2 (6%) |
| Scleral melting* | 2 (6%) |
| Corneal decompensation | 2 (6%) |
| Second primary tumour | 1 (3%) |
| Visual hindrance by the treated eye | 1 (3%) |
| Vascular occlusion | 1 (3%) |
| Sick tumour syndrome | 1 (3%) |
| Unknown (performed elsewhere) | 1 (3%) |

*Reasons for enucleation in enucleated patients (n=32). Multiple reasons can be registered for one patient.*

** The scleral doses of the two patients that had an enucleation due to scleral melting were 1068 Gy-equivalent and 1190 Gy-equivalent*

*Supplemental table 3: Risk factors for local failure after Ruthenium-106 brachytherapy*

| **Covariate** | **Hazard ratio** | **Confidence interval** | **p-value** |
| --- | --- | --- | --- |
| Juxtapapillary location | 4.906 | 2.666 – 9.028 | <0.001 |
| T-stage  T 1 (reference)  T 2  T 3-4 | 2.922  4.541 | 0.898 – 4.839  1.097 – 9.106 | 0.087  0.033 |
| Apex dose (per Gy-equivalent) | 0.971 | 0.945 – 0.997 | 0.032 |

*Regression table for risk of local failure in the first five years after therapy, corrected for treatment year. These covariates were chosen based on the shown correlation of juxtapapillary location [12, 17], tumour height[13, 38] and basal diameter[10, 12, 14-16] – which are both represented in the T-stage – and tumour dose[13, 17, 38] with local failure, combined with their biologically plausible connection with local failure.*
